# Single-Nanowire Fuse for Ionization Gas Detection

**DOI:** 10.3390/s19204358

**Published:** 2019-10-09

**Authors:** Hai Liu, Wenhuan Zhu, Yutong Han, Zhi Yang, Yizhong Huang

**Affiliations:** 1 Key Laboratory of Advanced Display and System Applications of Ministry of Education, Shanghai University, 149 Yanchang Road, Shanghai 200072, China; hliu5@shu.edu.cn; 2 National Key Laboratory of Science and Technology on Micro/Nano Fabrication, Shanghai Jiao Tong University, Shanghai 200240, China; zwhwin@sjtu.edu.cn; 3 Key Laboratory of Thin Film and Microfabrication of Ministry of Education, Shanghai Jiao Tong University, Shanghai 200240, China; sjtuhyt@sjtu.edu.cn (Y.H.); zhiyang@sjtu.edu.cn (Z.Y.); 4 School of Materials Science and Engineering, Nanyang Technological University, 50 Nanyang Avenue, Singapore 639798, Singapore

**Keywords:** Nano-electromechanical systems, nanosensor, single nanowire device, gas detectors

## Abstract

Local electric field enhancement is crucial to detect gases for an ionization gas sensor. Nanowires grown collectively along the identical lattice orientation have been claimed to show a strong tip effect in many previous studies. Herein, we propose a novel ionization gas detector structure by using a single crystalline silicon nanowire as one electrode that is placed above the prepatterned nanotips. A significant improvement of the local electric field in its radical direction was obtained leading to an ultralow operation voltage for gas breakdown. Different from the tip of the nanowire in the reported ionization gas sensors, the gaseous discharge current in this device flows towards the sidewall in the case of a trace amount of gas environment change. Technically, this discharge current brings about a sudden temperature rise followed by a fusion of the silicon nanowire. Such unique fusibility of a single nanowire in this gas detection device suggests a novel architecture that is portable and in-site executable and can be used as an integrated gas environmental monitor.

## 1. Introduction

Single nanowires have become a popular component in miniaturized optics, electronics or sensing devices [1,2,3,4,5,6]. The large specific surface, quick gaseous diffusion and ultralow energy consumption allows a single nanowire to play a key role in a chemical gas sensor [7,8,9,10,11], although realistic gas sensors, such as the electronic nose [12,13,14], fail to utilize the individual one-dimensional nanomaterials. A field ionization gas detector (FIGD) for instance, has been developed and is capable of differentiation of the gas types or concentrations by measuring the gas breakdown electric field. This specific ionization energy provides characteristic fingerprinting of gases. In order to lower the required voltage bias, multiple nanomaterials are collectively grown on the electrode pair including W, ZnO, Au, Si or carbon nanotubes [15,16,17,18,19]. These one-dimensional nanomaterials enhance the electric field towards their axis direction with a geometry effect. However, it is challenging to introduce a single nanowire into an FIGD.

In the present work, we successfully assembled a unique FIGD using a single nanowire as an electrode. In this device, the supplementary electric field enhancement is attributed to the scale effect in the radial direction of the silicon nanowire rather than the conventional axial direction. This exploratory architecture is the first device assembled that makes use of a single nanowire as a “fuse” during gas discharge. Such a controllable sensor provides a new route and a new technique for gas environmental detection and monitoring.

## 2. Materials and Methods

**Figure 1:**
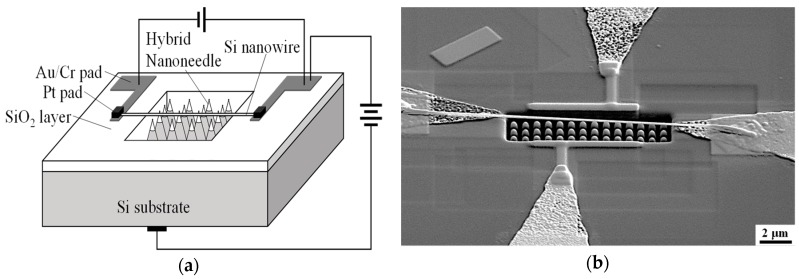
The structure of the ionization gas detection device consisting of a hybrid nanoneedles array and a single Si nanowire: (**a**) a schematic diagram and (**b**) a secondary electron image viewed at an oblique angle of 52°.

Figure 1a illustrates the schematic diagram of the FIGD setup, where a single nanowire is integrated into a couple of circuit loops: (1) a driving voltage is loaded between the silicon substrate and the nanowire for gas discharge, (2) a constant bias is applied on both terminals of the nanowire electrode through the Au/Cr pads to monitor the conductivity of the nanowire. A secondary electron image of this device was captured by a scanning electron microscope (SEM) and is shown in Figure 1b.

**Figure 2:**
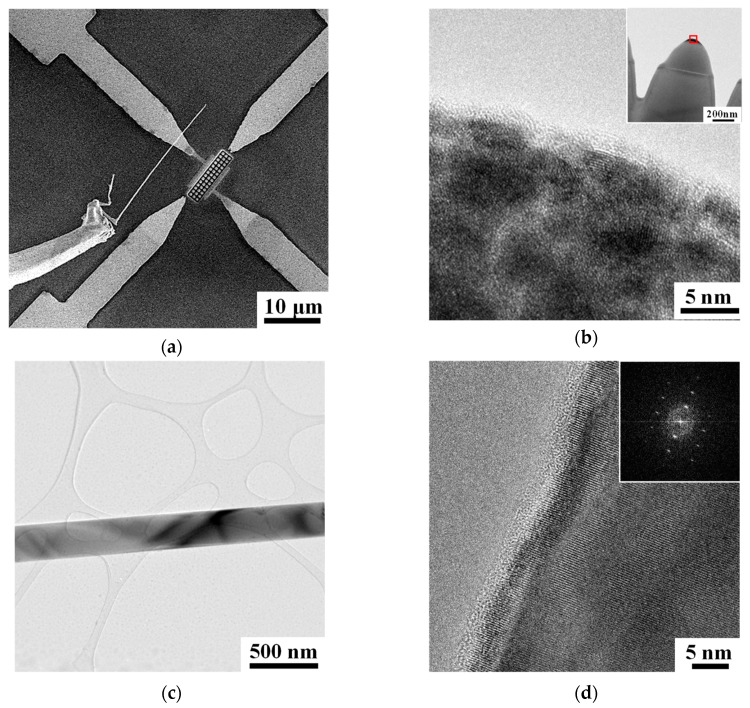
(**a**) A single nanowire is transferred to the top of nanoarray by a manipulator. (**b**) A high-resolution TEM image taken from the region marked in the inset of one hybrid nanoneedle, showing the dispersed Pt nanocrystallites in the amorphous matrix. (**c**) A bright field TEM image of a single silicon nanowire. (**d**) A high-resolution TEM image taken from the sidewall edge of nanowire in (**c**) with the inset showing its Fourier transform pattern.

**Figure A1:**
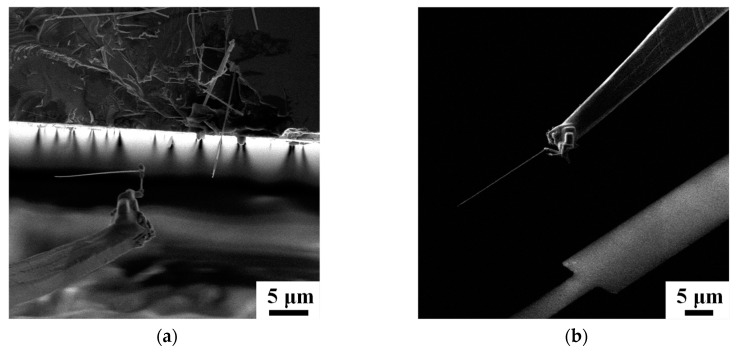
SEM images of a single silicon nanowire manipulation: (**a**) a single silicon nanowire was plucked away from the substrate by a manipulator, where the ion-assisted Pt deposition, a conventional technique of TEM sample preparation in the FIB system, allows their bonding; (**b**) the nanowire was transferred from the silicon substrate for nanowire growth to the pre-patterned SiO_2_ substrate.

This nanodevice was assembled by a hybrid nanoneedles array engraved in a SiO_2_/Si substrate followed by placing a single silicon nanowire on the top. The nanoneedles were fabricated in a focused ion beam system (FIB, FEI Nova Nanolab 600i) by ion meshing on the platinum-coated silicon substrate, as detailed in our previous papers [20,21,22,23]. First, a thin platinum stripe was deposited by the gas injection system (GIS) of the FIB on the surface of a SiO2 substrate (~280 nm oxide layer). The thickness (300 nm) and region (7 μm × 4.5 μm) of the Pt stripe can be precisely controlled by the ion beam irradiation duration (15 min) and the same scanned area, respectively. After that, an ion beam with energy of 30 keV and current of 2.8 nA was perpendicularly scanned on the SiO_2_ substrate in the same FIB system. The scan route was defined as a square mesh with an interval of 700 nm, which outlined the size and adjacent distance of the silicon nanoneedles in the array accordingly. The profile of each nanoneedle, especially the size of the nanotip, was monitored by in-situ secondary electron imaging during the ion beam irradiation. The silicon nanowires were grown on a conventional silicon substrate using a chemical vapor deposition (CVD) method, with dispersed gold colloids as catalyst and SiCl_4_ as the precursor [24]. One of the silicon nanowires was plucked (as detailed in Figure A1), transferred, and placed across the array above by a manipulator equipped in the FIB system (Figure 2a). The nanowires were in-situ welded to the prepatterned Au/Cr pads with a Ga^+^- assisted Pt deposition for the electrical connection [25].

## 3. Results

The microstructure of the nanowire and nanoneedle in this gas sensing device was characterized in a 200 kV transmission electron microscope (TEM, JEOL 2100F). The nanoneedles were retrieved from the array through the lift-out technique and thinned by the ion polishing process available in the FIB system [26]. As the high resolution TEM image shows in Figure 2b, many nanocrystallites with an extremely small size (~5 nm) are separately embedded into the amorphous matrix forming a nanodot that sits on top of the hybrid nanoneedle (inset to Figure 2b). The nanodot is measured as having a bottom diameter less than 100 nm and a height lower than 50 nm. There is a layer of insulating silicon dioxide between this conductive Pt nanodot and the lower silicon substrate with its original thickness of 280 nm. The diameter of the silicon nanowire is less than 300 nm as measured in Figure 2c, which is essential to the scaling effect for local electric field enhancement and current carrying capacity. Figure 2d shows the atomic structure of the nanowire around the sidewall with a higher magnification. As proven by the Fourier transform pattern (inset to Figure 2d), the silicon nanowire presents mainly in the form of a single crystal. 

**Figure 3:**
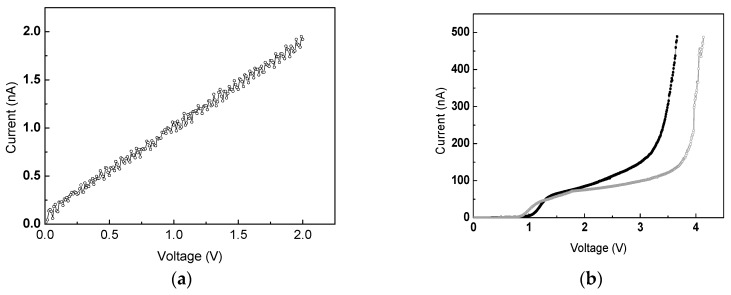
The electric measurement of the ionization gas detection device: (**a**) current versus voltage curve recorded by loading bias on both terminals of the single nanowire; (**b**) *I*-*V* data captured by sweeping voltage between the nanowire and substrate in the gas environment of N_2_ (grey) and NO_2_ diluted in N_2_ (black).

The electrical conductivity of the crystalline silicon nanowire was measured by a precision semiconductor parameter analyzer (Agilent 4156C). A typical current versus voltage (*I-V*) curve is plotted in Figure 3a, where the voltage bias ranges from 0 to 2 V in steps of 2 mV. The contact of the metallic pads with the silicon nanowire exhibits an ohmic conductance. This is due to the unique bonding technique in the FIB system using gallium ion irradiation during the formation of the platinum electrode, which brings about heavy p-type doping of Ga into the Si nanowire [27,28]. Meanwhile, the carrier transport capacity of this Si nanowire is measured to be much higher than the intrinsic one. It is believed that the Ga^+^ irradiation during device fabrication also results in light p-type doping to the overall nanowire to improve the electric conductivity, which is consistent with the results from reported doped single silicon nanowires [29,30,31].

Our previous work reported that the hybrid nanoneedles array reduces the ionization voltages [20,21]. In the present work, we introduced a single nanowire as the counter electrode, which exhibits several unique advantages over the hybrid nanoneedles including: (1) it allows the gas flow crossing the extremely narrow discharge space (submicron herein) to flow freely, which is hardly possible for the traditional plate-electrode sensor; (2) the diameter of the nanowire is in the order of hundreds of nanometers, which provides a local electrical field convergence effect allowing gaseous ionization at an ultralow voltage; (3) the nanowire suspended above the nanoneedles in the air is subject to fusion locally due to its low thermal conductivity but the high discharge current density in a certain gaseous electronics scenario [32,33,34,35] can function as a special “fuse” for monitoring the change of gas environment. It is believed that the temperature subjected to fusion suffers a rapid temperature rise, which is estimated to be up to 10^3^ K order of magnitude in a millisecond [34,35], as detailed in Appendix B.

In order to evaluate the properties of this new device, an example of detecting a trace of NO_2_ in a background of N_2_ was performed in this work. The device was placed into nitrogen initially and loaded by a sweeping voltage bias in order to generate a discharge current. Then the gas environment was changed to nitrogen dioxide diluted in the nitrogen with a concentration as low as 100 ppb for repeating gas discharge procedures. The typical *I-V* curves of the gas discharge are plotted in Figure 3b, where a current compliance of 500 nA was set for overcurrent protection. The current remains very low with the gradual growth of applied voltage but dramatically increases when the gas breakdown occurs. The breakdown threshold voltages are measured to be as low as 4 V for N_2_ and 3.5 V for 100 ppb NO_2_/N_2_ respectively. The *I-V* characteristics of gaseous electronics in different gases are directly determined by: (1) the charge distribution function, *F_c_*(*r*, *t*), (2) the field distribution function, *F_E_*(*r*, *t*), and (3) the properties of drift, convective, and diffusion processes of the resulting plasmas. All these factors result from some complicated processes of charge separation and transportation. Among those, the discussion of the previous literatures has been mainly focused on the role of the impact of a single nanostructure on the charge separation of gases [15,16,17,18,19]. In this work, the electrode system with nanostructures on both sides is governed by the polarity effect on the gases. Such a gaseous electronic scenario could be properly described by a one-dimensional simplification. Based on this hypothesis, we can qualitatively illustrate the basic observations in Figure 3b, supported by equation:

(1) $$I\left(t_{0}\right)=\int_{0}^{d}F_{c}\left(z,t_{0}\right)\left[F_{E}^{L}\left(z,t_{0}\right)+F_{E}^{s}\left(z,t_{0}\right)\right]\sigma \left(z,t_{0}\right)dz,$$

where the discharge current at specific time, $I\left(t_{0}\right)$, is given by the knowledge of $F_{c}$, the Laplacian field distribution function, $F_{E}^{L}$, and the superposed distribution function due to the space charge effect, $F_{E}^{s}$; *σ* is a measure of the drift mobility, and *d* is the electrode separation along the *z* axis normal to the nanoneedles array surface. Thus, for every single gas’ *I-V* curve after the ignition, due to the increase of both $F_{c}$ and $F_{E}^{L}$, higher *V* will give rise to higher *I*. For different gases, the individual gas’ *I-V* curve is initially governed by $F_{c}$ since any gas undergoes charge separation processes and shows a different charge capacity. Subsequently, the drift mobility of gas ions (*σ*) together with $F_{c}$ determines the space charge distribution ($F_{E}^{s}$), which is a strong function of the charge transport coefficient. In Figure 3b, the major difference between the pure nitrogen and NO_2_–N_2_ mixture is the ignition criterion, *V_c_*. Based on the model above, the formation of field-induced excitation/metastable population is due to the collective effect, which could be illustrated as follows: (1) The asymmetric setup of hybrid nanoneedles array/nanowire provides a strong polarization field, which increases the probability of field excitation, resulting in an increase of the metastable and excitation population, for the two gases (N_2_ and NO_2_). Since some nitrogen molecules have metastable energy levels higher than some ionization potential levels of NO_2_ species, e.g., N_2_ (A_3_Σ_u_), ~6.21 eV [36], Penning processes between NO_2_ and nitrogen may occur. Even at a low NO_2_ concentration, such processes can strongly affect the charge separation balance in the discharge system. Thus, an obvious decreasing *V_c_* can be expected due to the trace concentration of NO_2_.

**Figure 4:**
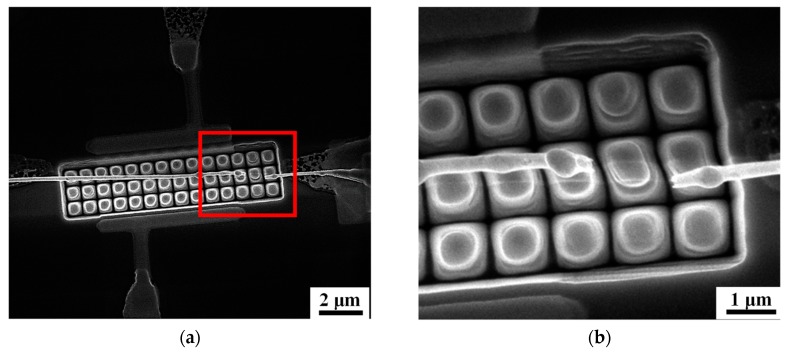
SEM images of nanowire failure: (**a**) a broken nanowire after gas discharge in NO_2_/N_2_; (**b**) the detailed morphology of the fractures in the region marked in (**a**).

After initializing the discharge fingerprints of the gas conditions, a constant bias between the different threshold voltages, 3.75 V, is selected as the operation voltage for monitoring. By applying this median voltage on the FIGD device, the occurrence of discharge is avoided in nitrogen, which is verified by the consistent nanowire conductivity measurement and microscopy observation. However, the gaseous breakdown occurs in the same device if the environment is replaced by 100 ppb NO_2_/N_2_, as confirmed from the SEM images in Figure 4. As shown in Figure 4a, without compliance protection, the failure occurs in the vicinity of the nanowire terminal connected to the Au/Cr pad of high potential. The fracture region is visible in Figure 4b, where the spherical droplet is seen to be formed over the first column of hybrid nanoneedles (the right couple of nanoneedles is platinum free, defined by the prepatterned Pt strip) [20]. The right droplet, however, is about two columns away from the Pt nanodots.

## 4. Discussion

**Figure 5:**
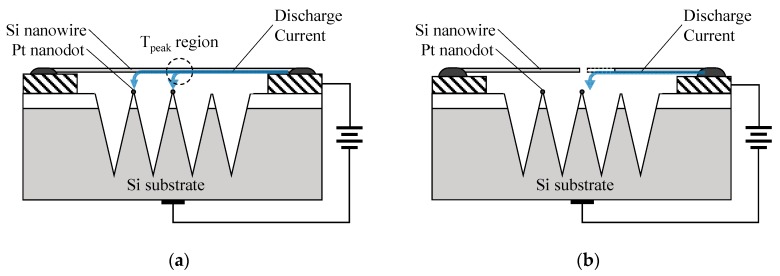
Schematic evolution of the nanowire failure in gas discharge: (**a**) gas breakdown current forming a concentrated high temperature region in the nanowire by Joule heating and (**b**) the local fusion of nanowire causes a fracture, where the remaining nanowire further shrinks along the subsequent discharge current flow to the nanotip.

The positions of the droplets imply that the fuse characteristic of the nanowire can be attributed to the discharge induced thermal melting, as two successive procedures illustrated in Figure 5. First, a discharge occurs between the nanowire and platinum nanodots rather than bare SiO_2_ nanoneedles as schematically shown in Figure 5a. Such spatial preference of gas ionization is attributed to the geometry and charge accumulation effect of the unique nanocrystallites, which is consistent with previous reports [20,21]. The joule heat provided by the discharge current throughout the nanowire is necessary to be considered. The nanowire in our device is homogeneous, and its electromigration or corrosion can be neglected. Therefore, a one-dimensional electrothermal field per volume can be described by the equation:

(2) $$k{\left(\frac{I}{A}\right)}^{2}=\rho c\times \frac{\partial T}{\partial t},$$

where *k* is the electric resistivity of the nanowire (7.07 × 10^−2^ Ω∙cm), the current *I* can be underestimated as the compliance (500 nA) over the cross-sectional area of the nanowire *A* (7.07 × 10^−10^ cm^2^), *ρ* is the mass density (2.3290 g/cm^3^) and *c* is the specific heat (703 J/kg/K) of the nanowire respectively, *T* is the temperature and *t* is the time. Then the temperature change rate to time can be estimated as 2.16 × 10^4^ K/s.

It was reported that the Joule heating causes an immediate fusion of nanoscale Au lines with high current density [37]. Similarly, the Si nanowire herein provides a further enhanced electric and thermal density [29,30,31,32], but low heat dissipation efficiency. Therefore, the fusion occurs in the Tpeak region resulting in droplet formation, as demonstrated by the morphology of the fracture shown in Figure 4.

**Figure A2:**
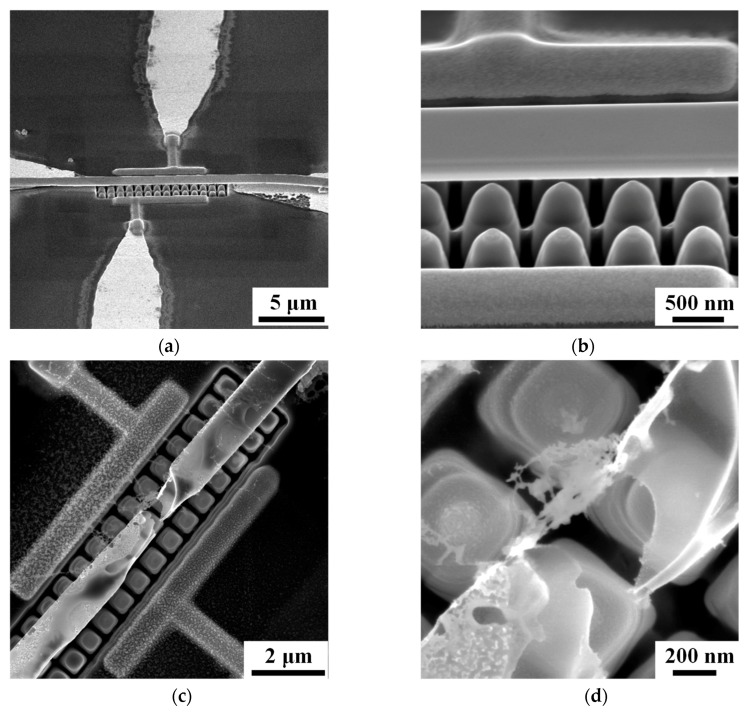
SEM images of an ionization gas detection device using a GaP nanoribbon and a nanoarray before (**a**,**b**) and after (**c**,**d**) gas discharge: (**a**) a single Gap nanoribbon is placed above the hybrid nanoneedles, which is detailed in (**b**), the metallic nanodot sitting on the top of each nanoneedle; (**c**) the breakdown of GaP nanoribbon and (**d**) the morphology characteristics of the fracture, where the products bridge the nanoribbon and nanodot with the expected electric field distribution.

As shown in Figure 5b, the tip of the broken nanowire terminal, instead of the nanowire sidewall, sustains the gas discharge and fusion as long as the gap becomes sufficiently large. Then, an extended gap between the nanowire fractures can be observed in Figure 4b and an open signal can be stably captured by the external circuit. It is worth noting that similar properties were also observed in these devices using other nanomaterials, such as gallium phosphide nanoribbon shown in Figure A2, which implies that a single nanomaterial fuse for FIGD is ubiquitous in the field of gaseous electronics.

## 5. Conclusions

In this work, a single silicon nanowire was used as the counter electrode in an FIGD. The nanowire was successfully assembled where a single nanowire was used as one of electrodes. It was found capable of tremendous enhancement of the local electrical field for a more effective discharge at the operation voltage. Importantly, by loading a proper voltage bias, this single nanowire is a fused subject to a subtle gas environment change. Through the gas sensing experiments, electrical tests, microstructure characterization, and nanowire failure analysis, we attributed the single-nanowire fuse to its electrothermal field induced by the discharge current. This nanowire–nanoneedle architecture explored a new route to realize ionization at an ultralow voltage, which is a difficult but important research topic in the field of gaseous electronics. This unique nano-micro device brings about a great potential for gas sensing applications, in various perspectives. For example, it can practically be used as environmental monitor/alarm, e.g. the internet of things (IoT).

## Appendix A

**Silicon nanowire manipulation**

## Appendix B

**Estimation of the temperature rise caused by gas discharge**

Based on the previous research about gas–solid thermal transport due to gas discharge [34], the temperature rise herein can be roughly estimated as:

$$T\left(t\right)=\alpha \frac{\beta UI}{nVC}t$$

where,

*α*: thermal accommodation coefficient from the ionized gas molecules with a mass density *n* to the nanowire, 2% [35]
*β*: the fraction of energy injected to the gas discharge space from the external circuit, 0.5 [36]
*U*: voltage loaded between the nanowire and the substrate, 3.75 V
*I*: current intensity in the circuit loop, 500 nA (underestimated)
*n*: gas density in the discharge space, for nitrogen in the standard environment, 1.2504 g/L
*V*: volume of the core discharge space, 300 nm × 2.4 μm × 300 nm = 2.16 × 10^−18^ m^3^
*C*: gaseous specific heat, for nitrogen, 1.038 J/g/K

The temperature rise caused by the gas discharge can be calculated as *T*(*t*) = 6.69 × 10^6^∙*t*, which means that the segment of nanowire in the vicinity of the Pt nanodot can be heated up to 10^3^ K order of magnitude (melting point of silicon) in around 1 millisecond.

## Appendix C

**A gas detection device of the same architecture using GaP nanoribbon**

## References

[1] Duan X., Huang Y., Agarwal R., Lieber C.M. (2003). Single-nanowire electrically driven lasers. Nature.

[2] Liu Z., Zhan Y., Shi G., Moldovan S., Gharbi M., Song L., Ma L., Gao W., Huang J., Vajtai R. (2012). Anomalous high capacitance in a coaxial single nanowire capacitor. Nat. Commun..

[3] Lupan O., Chow L., Pauporté T., Ono L.K., Cuenya B.R., Chai G. (2012). Highly sensitive and selective hydrogen single-nanowire nanosensor. Sens. Actuators B Chem..

[4] Zhang D., Liu Z., Li C., Tang T., Liu X., Han S., Lei B., Zhou C. (2004). Detection of NO_2_ down to ppb Levels Using Individual and Multiple In_2_O_3_ Nanowire Devices. Nano Lett..

[5] Moorthy B., Baek C., Eun Wang J., Jeong C.K., Moon S., Park K.-I., Kim D.K. (2017). Piezoelectric energy harvesting from a PMN–PT single nanowire. RSC Adv..

[6] Seo M.-H., Yoo J.-Y., Choi S.-Y., Lee J.-S., Choi K.-W., Jeong C.K., Lee K.J., Yoon J.-B. (2017). Versatile Transfer of an Ultralong and Seamless Nanowire Array Crystallized at High Temperature for Use in High-Performance Flexible Devices. ACS Nano.

[7] Das S.N., Kar J.P., Choi J.-H., Lee T.I., Moon K.-J., Myoung J.-M. (2010). Fabrication and Characterization of ZnO Single Nanowire-Based Hydrogen Sensor. J. Phys. Chem. C.

[8] Im Y., Lee C., Vasquez R.P., Bangar M.A., Myung N.V., Menke E.J., Penner R.M., Yun M. (2006). Investigation of a Single Pd Nanowire for Use as a Hydrogen Sensor. Small.

[9] Singh N., Yan C., Lee P.S. (2010). Room temperature CO gas sensing using Zn-doped In_2_O_3_ single nanowire field effect transistors. Sens. Actuators B Chem..

[10] Shao F., Hoffmann M.W.G., Prades J.D., Morante J.R., López N., Hernández-Ramírez F. (2013). Interaction Mechanisms of Ammonia and Tin Oxide: A Combined Analysis Using Single Nanowire Devices and DFT Calculations. J. Phys. Chem. C.

[11] Lupan O., Cretu V., Postica V., Ahmadi M., Cuenya B.R., Chow L., Tiginyanu I., Viana B., Pauporté T., Adelung R. (2016). Silver-doped zinc oxide single nanowire multifunctional nanosensor with a significant enhancement in response. Sens. Actuators B Chem..

[12] Gancarz M., Nawrocka A., Rusinek R. (2019). Identification of Volatile Organic Compounds and Their Concentrations Using a Novel Method Analysis of MOS Sensors Signal. J. Food Sci..

[13] Rusinek R., Gancarz M., Krekora M., Nawrocka A. (2019). A Novel Method for Generation of a Fingerprint Using Electronic Nose on the Example of Rapeseed Spoilage. J. Food Sci..

[14] Gancarz M., Wawrzyniak J., Gawrysiak-Witulska M., Wiącek D., Nawrocka A., Rusinek R. (2017). Electronic nose with polymer-composite sensors for monitoring fungal deterioration of stored rapeseed. Int. Agrophys..

[15] Singh J.P., Karabacak T., Lu T.-M., Wang G.-C., Koratkar N. (2004). Field ionization of argon using β-phase W nanorods. Appl. Phys. Lett..

[16] Yang W., Zhu R., Zong X. (2016). ZnO Nanowire-Based Corona Discharge Devices Operated Under Hundreds of Volts. Nanoscale Res. Lett..

[17] Sadeghian R.B., Kahrizi M. (2007). A novel miniature gas ionization sensor based on freestanding gold nanowires. Sens. Actuators A Phys..

[18] Liao L., Lu H.B., Shuai M., Li J.C., Liu Y.L., Liu C., Shen Z.X., Yu T. (2008). A novel gas sensor based on field ionization from ZnO nanowires: Moderate working voltage and high stability. Nanotechnology.

[19] Mohammadpour R., Ahmadvand H., Iraji zad A. (2014). A novel field ionization gas sensor based on self-organized CuO nanowire arrays. Sens. Actuators A Phys..

[20] Liu H., Yadian B., Liu Q., Gan C.L., Huang Y. (2013). A hybrid nanostructure array for gas sensing with ultralow field ionization voltage. Nanotechnology.

[21] Liu H., Wu J., Wang Y., Chow C.L., Liu Q., Gan C.L., Tang X., Rawat R.S., Tan O.K., Ma J. (2012). Self-Organization of a Hybrid Nanostructure consisting of a Nanoneedle and Nanodot. Small.

[22] Huang Y.Z., Wang S.G., Wang C., Xie Z.B., Cockayne D.J.H., Ward R.C.C. (2006). Self-organization of nanoneedles in Fe/GaAs (001) epitaxial thin film. Appl. Phys. Lett..

[23] Huang Y.-Z., Cockayne D.J.H., Ana-Vanessa J., Cowburn R.P., Wang S.-G., Ward R.C.C. (2008). Rapid fabrication of nanoneedle arrays by ion sputtering. Nanotechnology.

[24] Hochbaum A.I., Fan R., He R., Yang P. (2005). Controlled Growth of Si Nanowire Arrays for Device Integration. Nano Lett..

[25] Tham D., Nam C.-Y., Fischer J.E. (2006). Microstructure and Composition of Focused-Ion-Beam-Deposited Pt Contacts to GaN Nanowires. Adv. Mater..

[26] Kato N.I., Kohno Y., Saka H. (1999). Side-wall damage in a transmission electron microscopy specimen of crystalline Si prepared by focused ion beam etching. J. Vac. Sci. Technol. A Vac. Surf. Films.

[27] Tseng A.A. (2005). Recent Developments in Nanofabrication Using Focused Ion Beams. Small.

[28] Chattopadhyay S., Bohn P.W. (2004). Direct-write patterning of microstructured porous silicon arrays by focused-ion-beam Pt deposition and metal-assisted electroless etching. J. Appl. Phys..

[29] Chen H., Zou R., Chen H., Wang N., Sun Y., Tian Q., Wu J., Chen Z., Hu J. (2011). Lightly doped single crystalline porous Si nanowires with improved optical and electrical properties. J. Mater. Chem..

[30] Eranna G. (2014). Crystal Growth and Evaluation of Silicon for VLSI and ULSI.

[31] Cui Y., Duan X., Hu J., Lieber C.M. (2000). Doping and Electrical Transport in Silicon Nanowires. J. Phys. Chem. B.

[32] Li D., Wu Y., Kim P., Shi L., Yang P., Majumdar A. (2003). Thermal conductivity of individual silicon nanowires. Appl. Phys. Lett..

[33] Wu Y., Xiang J., Yang C., Lu W., Lieber C.M. (2004). Single-crystal metallic nanowires and metal/semiconductor nanowire heterostructures. Nature.

[34] Kondo K., Ikuta N. (1990). Spatio-Temporal Gas Temperature Rise in Repetitive Positive Streamer Corona in Air. J. Phys. Soc. Jpn..

[35] Bastien F., Marode E. (1985). Breakdown simulation of electronegative gases in non-uniform field. J. Phys. D Appl. Phys..

[36] Kirillov A.S. (2010). Electronic kinetics of molecular nitrogen and molecular oxygen in high-latitude lower thermosphere and mesosphere. Ann. Geophys..

[37] Wang M., Zhang B., Zhang G.P., Liu C.S. (2009). Evaluation of thermal fatigue damage of 200-nm-thick Au interconnect lines. Scr. Mater..

